# A Systematic Review and Meta-analysis of the Impact of the COVID-19 Pandemic on Access to HIV Pre-exposure Prophylaxis: Lessons for Future Public Health Crises

**DOI:** 10.1097/QAI.0000000000003488

**Published:** 2024-10-07

**Authors:** Luh Putu Lila Wulandari, Srila Nirmithya Salita Negara, Yusuf Ari Mashuri, Siska Dian Wahyuningtias, I. Wayan Cahyadi Surya Distira Putra, Yanri W. Subronto, Riris Andono Ahmad, Hasbullah Thabrany, Rebecca Guy, Matthew Law, Mohamed Hammoud, Benjamin B. Bavinton, John Kaldor, Nicholas Medland, Marco Liverani, Ari Probandari, David Boettiger, Virginia Wiseman

**Affiliations:** a The Kirby Institute, University of New South Wales, Sydney, Australia;; b Center for Tropical Medicine, Faculty of Medicine, Public Health, and Nursing, Universitas Gadjah Mada, Yogyakarta, Indonesia;; c Faculty of Medicine, Universitas Sebelas Maret, Surakarta, Indonesia;; d Department of Internal Medicine, Faculty of Medicine, Public Health, and Nursing, Universitas Gadjah Mada, Yogyakarta, Indonesia;; e Department of Biostatistics, Epidemiology, and Population Health, Faculty of Medicine, Public Health, and Nursing, Universitas Gadjah Mada, Yogyakarta, Indonesia;; f Faculty of Public Health, Universitas Indonesia, Jakarta, Indonesia;; g The United States Agency for International Development's (USAID) Health Financing Activity (HFA), Jakarta, Indonesia;; h Department of Global Health and Development, London School of Hygiene & Tropical Medicine, London, United Kingdom;; i School of Tropical Medicine and Global Health, Nagasaki University, Nagasaki, Japan; and; j Faculty of Public Health, Mahidol University, Bangkok, Thailand.

## Abstract

**Background:**

The World Health Organization is committed to strengthening access to pre-exposure prophylaxis (PrEP) for HIV prevention and its integration into primary care services. Unfortunately, the COVID-19 pandemic has disrupted the delivery of primary care, including HIV-related services. To determine the extent of this disruption, we conducted a systematic review and meta-analysis of the changes in access to PrEP services during the pandemic and the reasons for these changes.

**Methods:**

A search was conducted using PubMed, Scopus, Embase, PsycINFO, and Cinahl for studies published between January 2020 and January 2023. Selected articles described self-reported disruptions to PrEP service access associated with the COVID-19 pandemic or its responses. Pooled effect sizes were computed using a random-effects model.

**Results:**

Thirteen studies involving 12,652 PrEP users were included in our analysis. The proportion of participants reporting a disruption in access to PrEP services during the COVID-19 pandemic ranged from 3% to 56%, with a pooled proportion of 21% (95% confidence intervals: 8% to 38%). Social restrictions, financial constraints, and limited health insurance coverage were key factors affecting access to PrEP services during the pandemic.

**Conclusions:**

To our knowledge, this is the first meta-analysis to quantify the extent of disruptions to accessing PrEP services because of the COVID-19 pandemic. To increase the ability of primary care services to maintain PrEP services during public health crises, a mixture of strategies is worth considering. These include multi-month PrEP prescriptions, telehealth services, deployment of peer support groups to provide a community-based service or home delivery, and provision of financial support interventions.

## Abstract 2

Supplemental Digital Content is Available in the Text.

## INTRODUCTION

Pre-exposure prophylaxis (PrEP) use prevents HIV acquisition.^1–4^ Because of this benefit, the World Health Organization (WHO) includes PrEP in a recommended package of prevention services, targeting key population groups.^5–8^ This recommendation was also echoed by the Joint United Nations Programme on HIV/AIDS with its commitment to ensure access to PrEP for 3 million people at high risk of HIV infection by 2020.^9^ Furthermore, in 2022, the WHO reaffirmed its dedication to enhancing PrEP access and its integration into primary care services.^10^

The WHO declared the COVID-19 pandemic a public health emergency of international concern in January 2020.^11^ By 31 March 2024, more than 775 million confirmed COVID-19 cases and 7 million COVID-19-related deaths were reported to the WHO.^12^ The pandemic's impact on health care services, including primary care, has been profound, and collateral effects have also been observed. Although efforts have been made to continue HIV care delivery throughout the COVID-19 pandemic, disruptions to regular HIV services have been seen in many parts of the world,^13–16^ including access to condoms,^17^ HIV testing,^17,18^ HIV treatment,^15,16^ and PrEP.^17,19–21^

Throughout history, health care systems have faced similar disruptions during emergencies and crises. Examples include disruptions during wars or armed conflicts,^22–24^ natural disasters such as flooding and earthquakes,^25–27^ and previous disease outbreaks such as Ebola.^28^ These experiences have underscored the vulnerability of underprepared health care systems during crises, offering invaluable lessons. In 2021, in response to the COVID-19 pandemic, the WHO heightened its commitment to building health systems that are resilient against future public health threats.^29^

Although PrEP substantially reduces HIV transmission, its effectiveness heavily relies on the user's ability to access it in times of need. The experiences of both service providers and users throughout the COVID-19 pandemic can provide insights into the extent of the disruptions and the strategies necessary to develop resilient primary health care services that can ensure the continuous delivery of PrEP even in times of crisis. To the best of our knowledge, no meta-analysis has assessed the extent to which the COVID-19 pandemic impacted users' access to PrEP services.

To address this evidence gap and inform the design of policies and interventions to protect and strengthen PrEP services in the face of future public health threats, this systematic review was undertaken. The objectives of this review and meta-analysis were to identify the extent of disruptions to PrEP service access during the pandemic from a user perspective as well as the reasons for disrupted access and for the discontinuation of PrEP intake.

## METHODS

### Study Design

A systematic review and meta-analysis were undertaken, based on a comprehensive search of 5 databases: PubMed; Scopus; Embase; PsycINFO; and Cinahl. The search terms used were a combination of keywords and Medical Subject Heading terms on HIV, PrEP, and COVID-19 (see Supplemental Digital Content 1, http://links.lww.com/QAI/C327).

### Study Selection

Studies were included if they were empirical research, based on a quantitative study design, and published between January 2020 and January 2023. Studies also needed to report on the impact of the pandemic by measuring self-reported disruptions to PrEP service access, referred to as self-reported difficulties or discontinuation of an appointment to receive a new or refill prescription, to obtain PrEP medicines, or more difficult access to PrEP services in general. No limitations were applied regarding language or country, whereas research letters, reviews, meta-analyses, and studies for which the full text was unavailable were excluded.

The protocol for this review was registered at PROSPERO CRD42022299312.

Records were managed in EndNote X9. All articles obtained from the databases were imported to Endnote X9 and Excel spreadsheet, with duplicates removed by L.P.L.W. L.P.L.W., D.B., S.N.S.N., Y.A.M., S.D.W., and I.W.C.S.D.P. then conducted title and abstract screening.

### Data Extraction

Five authors (D.B., S.N.S.N., Y.A.M., S.D.W., and I.W.C.S.D.P.) then independently conducted full-text screening and data extraction using a standardized abstraction form, each handling approximately 7 different articles. Training sessions were provided by L.P.L.W. to ensure familiarity with the form and its usage. The data extracted included study details (authors, publication year), study characteristics (country, study design, study participants, recruitment sites, data collection period), and quantitative data reporting disruptions to PrEP service access. To prevent inconsistencies, L.P.L.W. reviewed all articles included in the data extraction process. Discrepancies were resolved by discussion between L.P.L.W. and D.B.

All data were imported and analyzed using Stata version 14 (StataCorp. 2019. College Station, TX).

### Data Analysis

The pooled prevalence of PrEP users experiencing disruptions to PrEP service access was presented as the percentage of PrEP users self-reporting disruptions to PrEP service access among the total number of PrEP users in the study. The meta-analysis was conducted according to the DerSimonian and Laird method,^30^ with the pooled prevalence computed using a random-effects model to account for heterogeneity in the effect estimate.^30–32^ Statistical tests for heterogeneity do not perform well with pooled proportions and were therefore not conducted.^33^ However, the sources of heterogeneity were explored through visual inspection of forest plots and the investigation of outliers.^33^ Heterogeneity was also explored using subgroup analyses.

PRISMA guidelines were used to guide the review process and the development of the manuscript.^34^

### Quality Assessment

The Joanna Briggs Institute Critical Appraisal Tool for Systematic Reviews was used to appraise the quality of the research evidence.^35^ D.B., S.N.S.N., Y.A.M., S.D.W., and I.W.C.S.D.P. independently assessed the quality of the included studies. Any discrepancies were addressed through discussions with L.P.L.W. Each study underwent evaluation against the criteria outlined in the tool, resulting in categorization into quality tiers. Studies meeting over 80% of the tool's criteria were deemed high quality, whereas those meeting 50%–80% were considered moderate, and those meeting less than 50% were categorized as low quality. Studies meeting 50% or more of the criteria were eligible for inclusion. This process mirrors the methodology used in previous meta-analyses.^36^

## RESULTS

### Study Characteristics

**FIGURE 1.**
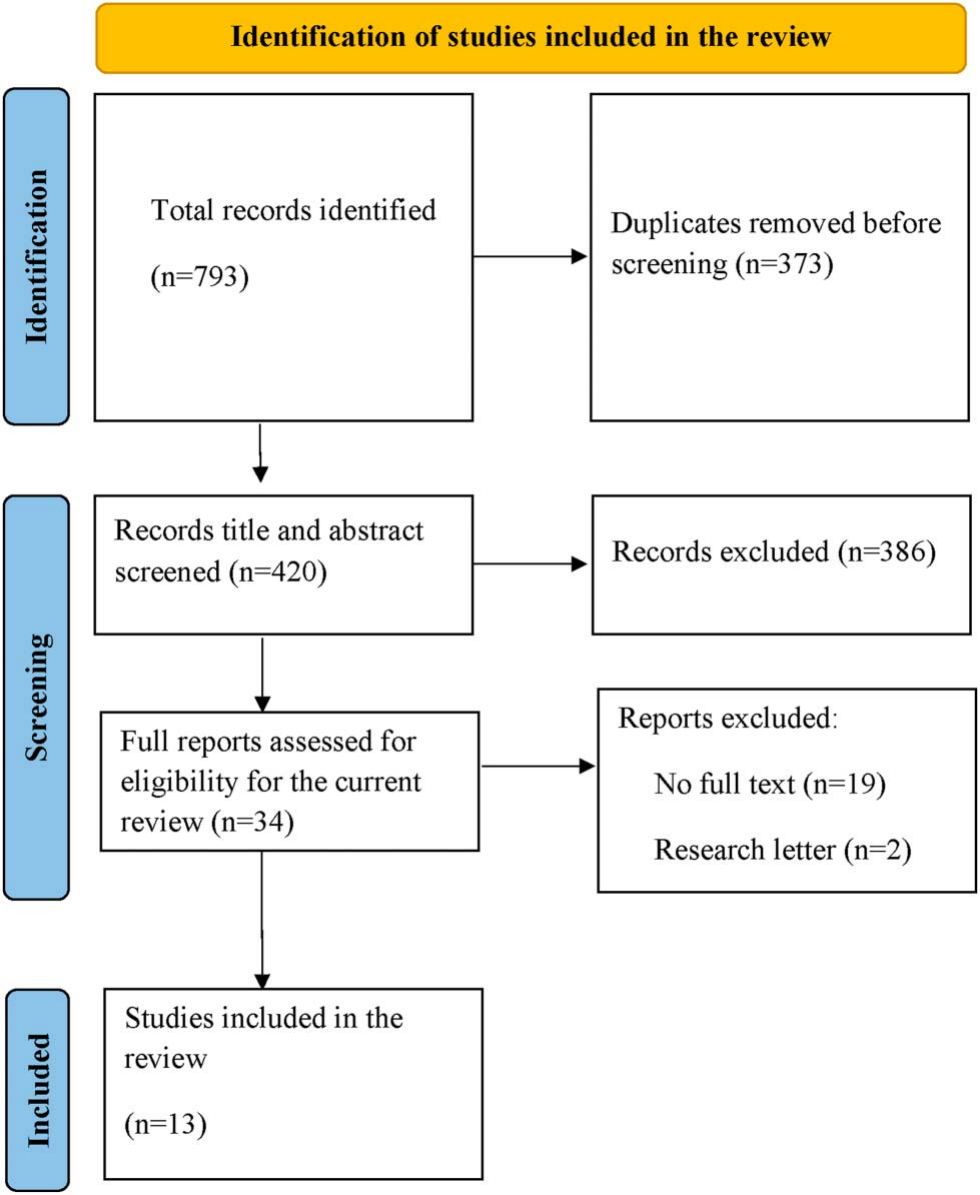
Study selection.

Our search found 793 articles. Among these, 373 duplicates were removed, and 386 were excluded after title and abstract screening. Of the 34 full reports reviewed, 19 were excluded because there was no full text, and 2 were research letters. These exclusions resulted in 13 studies eligible for analysis (Fig. 1). These 13 studies were conducted in 19 countries. Specifically, 12 studies were conducted within a single country—comprising 1 middle-income^37^ and 11 high-income countries, as classified by the World Bank's income classification.^38^ In addition, 1 study was conducted across 20 countries, encompassing 11 middle-income and 9 high-income countries^17^ (Table 1).

**TABLE 1.** Characteristics of Studies Included in the Analysis

Authors	Publication Year	Country	World Bank's Country Income Classification for the 2024 Fiscal Year	Study Design	Data Collection Methods	Data Collection Period	Data Collection Period in Relation to Government-Imposed Restriction Periods	Study Population(s)	Sampling Strategy	Recruitment Sites
Camp et al^39^	2021	The United States	High-income country	Cross-sectional	Email survey	December 2019 and June 2020	During restriction	MSM	Nonprobability	Clinic/s and social media or web-based
Chen et al^20^	2021	The United States	High-income country	Cross-sectional	A Zoom platform interview	April–July 2020	During restriction	MSM and transgender women	Probability	Community
Ciaccio et al^40^	2022	France	High-income country	Cross-sectional	Online survey	June–July 2020	During restriction	MSM	Nonprobability	Social media or web-based
Hammoud et al^41^	2021	Australia	High-income country	Cross-sectional	Online survey	April 2020	During restriction	Gay and bisexual men	Nonprobability	Social media or web-based
Hong et al^18^	2021	The United States	High-income country	Cross-sectional	Online survey	April–September 2020	Unreported	Young sexual minority men (YSMM)	Nonprobability	Social media or web-based
MacCarthy et al^21^	2020	The United States	High-income country	Mixed-methods study	Telephone-based or app-based interview	April–May 2020	Unreported	Latin sexual minority men (LSMM) and transgender women (LTGW)	Probability	Non-govermental organisations
Mistler et al^42^	2021	The United States	High-income country	Cross-sectional	Telephone-based or app-based interview	May–October 2020	Unreported	PWIDs with opioid use disorder	Nonprobability	Clinic/s
Morgan et al^43^	2022	The United States	High-income country	Cohort	Online survey	March–August 2020	Unreported	Black and/or Hispanic/Latino sexual minority men and gender diverse (SMMGD)	Nonprobability	Social media or web-based
Pampati et al^44^	2021	The United States	High-income country	Cross-sectional	Online survey	October 2019–July 2020	Unreported	MSM	Unreported	Social media or web-based
Rao et al^17^	2021	Australia, Belarus, Belgium, Brazil, Canada, Egypt, France, German, Indonesia, Italy, Kazakhstan, Malaysia, Mexico, the Russian Federation, Taiwan, Thailand, Turkey, Ukraine, the United Kingdom, and the United States	Nine high-income countries and 11 middle-income countries	Cross-sectional	Online survey	April–May 2020	During restriction	Users of gay social networking app Hornet	Nonprobability	Social media or web-based
Reyniers et al^45^	2021	Belgium	High-income country	Cross-sectional	Online survey	April 2020	During restriction	MSM	Nonprobability	Social media or web-based
Stephenson et al^46^	2021	The United States	High-income country	Cross-sectional	Online survey	April–May 2020	During restriction	Gay, bisexual, and other MSM (GBMSM)	Nonprobability	Social media or web-based
Torres et al^37^	2021	Brazil	Middle-income country	Cross-sectional	Online survey	April–May 2020	During restriction	MSM and transgender/non-binary (TGNB)	Nonprobability	Social media or web-based

In total, 12,652 PrEP users were included in the studies. All studies were conducted as part of larger cross-sectional, cohort, or mixed-methods studies. Most studies (n = 11) used a cross-sectional study design,^17,18,20,37,39–42,44–46^ with only 1 using a cohort design^43^ and 1 a mixed-methods design.^21^ Most (n = 8) used an email or online survey, 2 used phone interviews,^21,42^ and 1 used a Zoom platform.^20^ Twelve studies recruited men who have sex with men (MSM) or lesbian, gay, bisexual, transgender and queer participants, with 6 studies specifically recruiting only MSM participants. One study was among people who inject drugs (PWIDs).^42^ Nine studies recruited participants from online networks or mobile apps and 4 studies recruited participants from clinics, nongovernmental organizations, or community settings. All studies collected their data around 2020, with 2 starting their data collection process in 2019.^39,44^ The period of data collection ranged from 1 to 9 months. Most studies (n = 10) were published in 2021, 1 in 2020, and 2 in 2022 (Table 1).

### Disruption in Access to PrEP Services During the COVID-19 Pandemic

**FIGURE 2.**
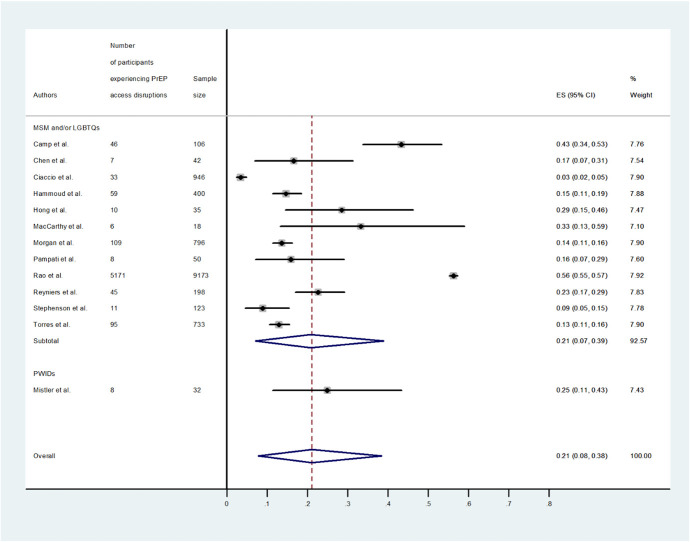
Forest plot of the proportion of PrEP users experiencing disruptions in access to PrEP services during the COVID-19 pandemic.

Among the 13 studies included in the meta-analysis, the proportion of participants self-reporting disruptions to PrEP service access ranged from 3% to 56%, indicating substantial heterogeneity, with an overall pooled proportion of PrEP users experiencing disruptions to PrEP service access of 21% [95% confidence intervals (CI): 8% to 38%] (Fig. 2).

Stratified by population groups, a study conducted among PWIDs (n = 1) found that 25% (95% CI: 11% to 43%) of PrEP users experienced disruptions to PrEP service access. The pooled proportion of disruptions among those self-identified as MSM or lesbian, gay, bisexual, transgender and queer (n = 12) was lower, that is, 21% (95% CI: 7% to 39%) (Fig. 2). Two studies explored the reasons for such disruptions.^20,21^ Among the factors impacting the changes in access to PrEP services included social restrictions,^21^ financial constraints,^20^ and health insurance issues.^21^

### PrEP Discontinuation and HIV Testing

In addition to disruptions to PrEP service access, some studies also reported user experiences of PrEP discontinuation, that is, interruptions in the use of PrEP (n = 5)^18,21,37,40,41^ and HIV testing disruptions (n = 8).^17,18,21,41,42,44–46^ Among the self-reported reasons for discontinuing PrEP use were reduced sexual activity or fewer sexual partners.^18,21,37,40,41^ One study explored the association between PrEP discontinuation and HIV testing and found that those who had discontinued PrEP had lower HIV testing rates compared with those who used PrEP during the COVID-19 pandemic.^41^

### Risk of Bias and Quality of the Studies Included in the Analysis

The quality of the studies included in the analysis is depicted in Supplemental Digital Content 2, http://links.lww.com/QAI/C327. The appropriateness of the sampling frame and the adequacy of the sample size were unclear in most studies, although this issue is common when recruiting hidden or stigmatized populations.^47^ All studies described settings and subjects in detail.

## DISCUSSION

Strengthening primary health care resilience to ensure access to PrEP and other HIV services is a priority for many countries. To our knowledge, this is the first meta-analysis to quantify the extent of disruptions to accessing PrEP services because of the COVID-19 pandemic, drawing attention to both high-income and low- and middle-income countries (LMICs), and inclusion of different key populations. We estimate the pooled proportion of PrEP users experiencing disruptions in access to be 21% (95% CI: 8% to 38%). This result is similar to the predicted reduction in PrEP prescriptions during the pandemic in the United States, modelled using a national pharmacy database, that is, 22% (95% CI: 19% to 25%).^48^ Disruptions to PrEP access increase vulnerability to HIV infection.^43^ Although some studies have reported a decrease in PrEP use because of a reduction in demand (ie, a decline in sexual contacts),^18,40^ some groups remained engaged in behaviors that could lead to HIV risk during the pandemic,^18^ including condomless sex^40,49^ and having multiple sexual partners.^50,51^

One of the key factors cited for disruptions to PrEP service access during the pandemic was financial barriers, including travel-related costs.^20^ Studies show that even before the COVID-19 pandemic, the cost of PrEP-associated consultations, medication, and laboratory tests was already a key barrier to accessing PrEP services, especially among socioeconomically disadvantaged or underinsured groups.^20,52–56^ In LMICs, it is common for patients to make substantial out-of-pocket payments toward their health care.^57^ These costs were exacerbated during the pandemic when many health services closed or reduced their patient intake, forcing patients to travel further to access PrEP.^20^ Given that many key populations, such as MSM or sex workers, lacked social support^58^ and experienced loss of income during the COVID-19 outbreak,^58,59^ financial support interventions, including cost-sharing, are expected to help maintain PrEP access during future public health crises.^60^ Further research is needed to investigate the effectiveness, cost-effectiveness and affordability of such interventions especially during public health crises.

PrEP clinical practice guidelines recommend that PrEP users undertake routine HIV testing.^61^ One study in Australia included in this review noted a strong correlation between the discontinuation of PrEP during COVID-19 restrictions and the lower likelihood of recently being tested for HIV.^41^ Similar findings were reported in the United States.^46^ A decrease in testing rates could be associated with a reduction in at-risk behaviors, which might consequently have a slight impact on the transmission of sexually transmitted infections. However, mathematical modeling has indicated that, despite a decrease in the number of sexual partners, the decline in HIV testing rates during the COVID-19 pandemic is linked to an increase in sexually transmitted infection transmission rates. This is especially notable for chlamydia trachomatis.^62^ Therefore, the disruption to PrEP service access, and thus access to HIV testing, underscores the need to consider alternative strategies to ensure the provision of testing services during public health crises.

In several countries, efforts were made to ensure sustainable access to PrEP during the COVID-19 pandemic. Initiatives included the provision of a multi-month PrEP supply,^63–65^ maintaining or extending clinic operating hours even during periods of social restriction,^66^ telehealth consultations,^64–67^ use of social media platforms to maintain demand and provide education about PrEP,^64^ additional community support for PrEP care (eg, peer support, lay health care providers,^3^ outreach workers^64,68^) and PrEP home delivery.^69^ Although the success of such telehealth interventions depends, for example, on good infrastructure and access to technology to enable telehealth,^70^ these interventions are shown to be feasible and effective across a range of settings and target groups.^64^

Our studies conducted in Indonesia highlighted several challenges encountered in mitigating the impact of the COVID-19 pandemic on health care systems while striving to sustain the delivery of HIV and tuberculosis (TB) services.^71,72^ These challenges encompassed interruptions in the supply of HIV and TB medicines, increased workload among health care workers tasked with managing both HIV and COVID-19-related strategies, and heightened risk of COVID-19 exposure among community health workers and peer groups of people living with HIV.^71,72^ It is imperative to consider these challenges when ensuring continued access to PrEP during public health crises.

A previous study showed that PrEP users strongly favored home delivery of PrEP and the use of HIV self-tests, supported by telemedicine services.^73^ Scaling up this delivery method is worth considering to ensure continued access and use of PrEP along with HIV testing during future crises.

Because of the enormous pressures on health systems during a pandemic and other public health emergencies, integration of PrEP into wider health services might also be worth considering to improve efficiency and ensure the continuation of PrEP. For example, as noted in some settings, a syringe services program could also be used to provide PrEP for PWIDs.^74,75^ Community-based antiretroviral delivery, involving outreach workers partnering with community-based organizations, was shown to have a positive impact on antiretroviral retention^76^ and might also be evaluated for use in other settings.

There are a few limitations to be considered when interpreting the results of this review. First, the current review included studies from only 19 countries, most of which were classified as high-income. Thus, the data presented are unlikely to be representative of LMICs more generally. Future studies would benefit from delving deeper into this issue within LMIC contexts. Second, study designs and methodologies used in each study varied widely. This could introduce variability, that is, clinical or methodological heterogeneities, which might then result in statistical heterogeneity.^32^ While conducting a statistical test to determine whether these variations are greater than what is expected by chance alone is important,^32^ the statistical tests for heterogeneity do not perform well with pooled proportions and were therefore not conducted.^33^ As a result, we were only able to explore heterogeneity using subgroup analyses and visual inspection. Thus, caution should be taken when interpreting the pooled estimate. Third, many studies lacked detailed information on timelines, making it difficult to determine whether data on disruptions to accessing PrEP services related to the lockdown period. Fourth, in our meta-analysis, the sample was drawn from various subsamples or was part of a larger study, which resulted in a lack of specificity regarding age, gender, and rural/urban location within the subsamples in the included studies. Fifth, reduced at-risk behavior may have led to a decline in PrEP need or a change from daily dosing to event-based dosing in MSM. This could have resulted in an overestimate of the level of disruption as some PrEP users who reported not being able to access PrEP services may not in fact have needed them. Finally, all studies used self-reported measures of disruptions to accessing PrEP services, and this may be prone to social desirability bias.^77^ Considering this limitation, future research on this topic could explore alternative methods, such as using health clinic visit data for PrEP appointment attendance, prescriptions, or refills. This approach may offer complementary evidence to generate a richer understanding of disruptions in PrEP service utilization.

## CONCLUSIONS

This systematic review and meta-analysis quantified the level of disruptions in access to PrEP services during the COVID-19 pandemic. Policymakers and health providers need to ensure that PrEP users can continue to access PrEP services during public health crises and emergencies. Strategies worthy of consideration include the provision of financial support interventions including cost-sharing, digital or multi-month prescription or supply of medicines, telehealth consultations, and PrEP home delivery, including the provision of HIV self-testing.

## Supplementary Material
