# Supplementary material for: A Systematic Review and Meta-analysis of the Impact of the COVID-19 Pandemic on Access to HIV Pre-exposure Prophylaxis: Lessons for Future Public Health Crises
Source: J Acquir Immune Defic Syndr. 2024 Oct 7;97(3):208–15. doi: 10.1097/QAI.0000000000003488 (PMC11458105; doi:10.1097/QAI.0000000000003488)
Supplement: Supplementary file 1 [file qai-97-208-s001.docx]

**Supplement 1. Search strategies: a combination of keywords and Medical Subject Heading (MeSH) terms on HIV, pre-exposure prophylaxis, and COVID-19**

| **Database** | **Concept terms** |
| --- | --- |
| Pubmed | (COVID-19 [Text Word])) OR (COVID 19 [Text Word])) AND (((HIV[MeSH Terms]) OR (HIV[Text Word]) OR (Human Immunodeficiency Virus[Text Word]) OR (HIV AIDS[Text Word])) AND ((Pre-Exposure Prophylaxis[MeSH Terms]) OR (Pre Exposure Prophylaxis[Text Word]) OR (Pre-Exposure Prophylaxi[Text Word]) OR (Prophylaxi, Pre-Exposure[Text Word]) OR (Prophylaxis, Pre-Exposure[Text Word]) OR (Pre-Exposure Prophylaxis (PrEP)[Text Word]) OR (Pre Exposure Prophylaxis (PrEP)[Text Word]) OR (Pre-Exposure Prophylaxi (PrEP)[Text Word]) OR (Prophylaxi, Pre-Exposure (PrEP)[Text Word]) OR (Prophylaxis, Pre-Exposure (PrEP)[Text Word]))) |
| Scopus | ( ( ( TITLE-ABS-KEY ( hiv ) OR TITLE-ABS-KEY ( "Human Immunodeficiency Virus" ) OR TITLE-ABS-KEY ( "HIV AIDS" ) ) ) AND ( ( TITLE-ABS-KEY ( "Pre-Exposure Prophylaxis" ) OR TITLE-ABS-KEY ( "Pre Exposure Prophylaxis" ) OR TITLE-ABS-KEY ( "Pre-Exposure Prophylaxi" ) OR TITLE-ABS-KEY ( prophylaxi, AND pre-exposure ) OR TITLE-ABS-KEY ( prophylaxis, AND pre-exposure ) OR TITLE-ABS-KEY ( "Pre-Exposure Prophylaxis (PrEP)" ) OR TITLE-ABS-KEY ( "Pre Exposure Prophylaxis (PrEP)" ) OR TITLE-ABS-KEY ( prep ) ) ) ) AND ( ( TITLE-ABS-KEY ( covid-19 ) OR TITLE-ABS-KEY ( "COVID 19" ) ) ) |
| Embase | 1 HIV.mp. or exp Human immunodeficiency virus/  2 Pre-Exposure Prophylaxis.mp. or exp pre-exposure prophylaxis/  3 Pre Exposure Prophylaxis.mp.  4 COVID-19.mp. or coronavirus disease 2019/  5 COVID 19.mp.  6 2 or 3  7 1 and 6  8 4 or 5  9 7 and 8 |
| APA PsycINFO | 1 HIV.mp. or exp Human immunodeficiency virus/  2 Pre-Exposure Prophylaxis.mp. or exp pre-exposure prophylaxis/  3 Pre Exposure Prophylaxis.mp.  4 COVID-19.mp. or coronavirus disease 2019/  5 COVID 19.mp.  6 2 or 3  7 1 and 6  8 4 or 5  9 7 and 8 |
| Cinahl | S1 MW hiv OR TX hiv OR TX "Human Immunodeficiency Virus" OR TX "HIV AIDS"  Expanders - Also search within the full text of the articles; Apply equivalent subjects  Search modes - Boolean/Phrase  S2 MW ( pre-exposure prophylaxis or prep or preexposure prophylaxis ) OR TX "pre-exposure prophylaxis" OR TX prep OR TX "preexposure prophylaxis"  Expanders - Also search within the full text of the articles; Apply equivalent subjects  Search modes - Boolean/Phrase  S3 (MW ( pre-exposure prophylaxis or prep or preexposure prophylaxis ) OR TX "pre-exposure prophylaxis" OR TX prep OR TX "preexposure prophylaxis") AND (S1 AND S2)  Expanders - Also search within the full text of the articles; Apply equivalent subjects  Search modes - Boolean/Phrase  S4 MW covid-19 pandemic OR TX COVID-19 OR TX "COVID 19"  Expanders - Also search within the full text of the articles; Apply equivalent subjects  Search modes - Boolean/Phrase  S5 (MW covid-19 pandemic OR TX COVID-19 OR TX "COVID 19") AND (S3 AND S4)  Expanders - Also search within the full text of the articles; Apply equivalent subjects  Search modes - Boolean/Phrase |

**Supplement 2. Critical appraisal of studies included in the analysis**

| Authors | Was the sample frame appropriate to address the target population? | Were study participants sampled in an appropriate way? | Was the sample size adequate? | Were the study subjects and the setting described in detail? | Was the data analysis conducted with sufficient coverage of the identified sample? | Were valid methods used for the identification of the condition? | Was the condition measured in a standard, reliable way for all participants? | Was there appropriate statistical analysis? | Was the response rate adequate, and if not, was the low response rate managed appropriately? | Quality assessment tier |
| --- | --- | --- | --- | --- | --- | --- | --- | --- | --- | --- |
| Camp et al.^39^ | Unclear | Yes | Unclear | Yes | Unclear | Yes | Yes | Yes | Unclear | Moderate |
| Chen et al. ^20^ | Unclear | Yes | Unclear | Yes | Yes | Yes | Yes | Yes | Yes | High |
| Ciaccio et al. ^40^ | Unclear | Yes | Unclear | Yes | Yes | Yes | Yes | Yes | Yes | High |
| Hammoud et al.^41^ | Unclear | Yes | Unclear | Yes | Yes | Yes | Yes | Yes | Yes | High |
| Hong et al.^18^ | Unclear | Yes | Unclear | Yes | Yes | Yes | Yes | Yes | Yes | High |
| MacCarthy et al.^21^ | Unclear | Yes | Unclear | Yes | Yes | Yes | Yes | Yes | Yes | High |
| Mistler et al.^42^ | Unclear | Yes | Unclear | Yes | Yes | Yes | Yes | Yes | Unclear | Moderate |
| Morgan et al.^46^ | Unclear | Yes | Unclear | Yes | Yes | Yes | Yes | Yes | Yes | High |
| Pampati et al.^43^ | Unclear | Unclear | Unclear | Yes | Yes | Yes | Yes | Yes | Yes | Moderate |
| Rao et al.^17^ | Unclear | Yes | Unclear | Yes | Yes | Yes | Yes | Yes | Yes | High |
| Reyniers et al.^44^ | Unclear | Yes | Unclear | Yes | Yes | Yes | Yes | Yes | Yes | High |
| Stephenson et al.^45^ | Unclear | Yes | Unclear | Yes | Yes | Yes | Yes | Yes | Yes | High |
| Torres et al.^37^ | Unclear | Yes | Unclear | Yes | Yes | Yes | Yes | Yes | Yes | High |
